# Dating and friendships in adolescence: Variation across same-sex and other-sex romantic partners

**DOI:** 10.1111/jora.12865

**Published:** 2023-05-21

**Authors:** Rose Wesche, Derek A. Kreager, Nayan G. Ramirez, Shivangi Gupta

**Affiliations:** 1 Virginia Polytechnic Institute and State University, Blacksburg, Virginia, USA; 2 The Pennsylvania State University, University Park, Pennsylvania, USA; 3 California State University Northridge, Northridge, California, USA

**Keywords:** dating, peer relationships, sexual minority youth

## Abstract

This research examined associations between dating and number of friends for rural adolescents with same-sex and other-sex dating partners using longitudinal sociometric data (*N* = 2826; 55% female, 87% White, mean age = 14 at baseline). In multilevel models assessing within-person change, boys gained female friends when they were in same-sex romantic relationships, compared to when they were single. In contrast, girls in same-sex relationships lost female friends and gained male friends. Adolescents in other-sex romantic relationships gained same-sex friends compared to when they were single. Results advance understanding of adolescent social and sexual development, suggesting that sexual minority adolescents find allies when dating but may struggle to maintain same-sex friendships.

Forming romantic relationships is an important developmental task of adolescence, but also one that likely differs substantially between heterosexual and sexual minority youth. For adolescents with other-sex partners, dating is associated with numerous positive outcomes, including increased social status (Furman et al., 2009; Furman & Rose, 2015; Miller et al., 2009; Ryjova et al., 2021; Savickaitė et al., 2019). However, the association between dating and peer relationships for adolescents with same-sex partners is unknown. Understanding how dating is associated with changes in the number of friendships for adolescents with same-sex partners will further elucidate the potential obstacles and opportunities of sexual minority adolescents embedded in school-based peer networks. This knowledge can inform the developmental understanding of dating and peer relationships among sexual minority youth. This knowledge can also support efforts to strengthen peer relationships of sexual minority youth.

## The peer context of adolescent dating for heterosexual adolescents

Romantic relationships represent an emergent and increasingly salient social tie in adolescence, distinguished from peer friendships by their intense emotions, high levels of intimacy, and the possibility of sexual behavior (Collins et al., 2009). Connecting romantic relationships to the peer contexts in which they emerge, script theory suggests that heterosexual adolescents may benefit socially from forming romantic relationships. Script theory proposes that individuals internalize, then enact, cultural messages about how we should interact with one another and form relationships (Patterson et al., 2013; Simon & Gagnon, 2003; Wiederman, 2015). Scripts related to dating and sexuality are part of a gendered heteronormative discourse that emphasizes heterosexual relationships as visible and socially desirable (Wiederman, 2015). Adolescents who successfully negotiate opposite-sex romantic partnerships should then be elevated within peer friendship networks. Consistent with this framework, cross-sectional and longitudinal research indicates that dating an other-sex partner improves adolescents’ social status. In cross-sectional research, adolescents with romantic partners tend to be more well-liked than their single peers (Furman et al., 2009; Furman & Rose, 2015; Miller et al., 2009; Savickaitė et al., 2019). In a longitudinal study, Ryjova et al. (2021) found that, for Asian American but not Hispanic adolescents, abstaining from dating was associated with worse future peer status.

Gender also plays a role in determining the associations between dating and friendships, particularly for heterosexual adolescents. Romantic relationships exist in the context of the sexual double standard, which rewards men’s sexual activity while punishing women’s (Sagebin Bordini & Sperb, 2013). Like sexual behavior, a separate double standard presides over adolescents’ romantic relationships, suggesting that romantic involvement is encouraged for boys but risky for girls and that the social consequences of dating may differ by gender (Moreau et al., 2019). Therefore, the association between dating and friendships may differ for boys versus girls.

## Dating and friendships for sexual minority adolescents

Past research on dating and peer acceptance has relied primarily on heterosexual samples. Dating is also an important developmental process for sexual minority adolescents. Recognition theory proposes that interpersonal and community recognition (i.e., love and social esteem) are critical requirements for self-realization, such as identity development (Honneth, 1996). For sexual minority adolescents, recognition can manifest as having strong peer networks, allyship from peers, and representation and visibility in their social settings (Ceatha et al., 2021). Forming romantic relationships, and being able to maintain friendships as they navigate romantic development, would indicate that sexual minority adolescents can foster resilience and find recognition as they grow. In addition to finding recognition, romantic relationships promote independence, sexual exploration, and the development of interpersonal skills (Connolly et al., 2023). Despite the developmental importance of dating, extant research provides little insight into how sexual minority adolescents navigate dating in their social contexts.

Sexual minority adolescents’ social landscape is complex and can contain both stressors and assets. These experiences have implications for associations between dating and number of friends. Regarding social stressors, stigma against same-sex attraction exists (Mink et al., 2014). This stigma may manifest as social rejection or victimization. For example, in one study of sexual and gender minority adolescents, those who were “out” at school experienced more peer victimization than those who concealed their identities (Russell et al., 2014). Other research also supports a link between sexual minority identity disclosure to peers and higher rates of peer victimization (Zhao et al., 2021). In general, sexual minority adolescents may have fewer friends than heterosexual adolescents and are less central in peer networks (la Roi et al., 2020; Ramirez et al., in press, but see Hatzenbuehler et al., 2012; Ueno, 2005). When dating a same-sex peer in school, adolescents may increase the visibility of their sexual identity and experience increased stigma, manifesting in fewer friendships.

Although some peers may react negatively to same-sex dating, other heterosexual youth may support such ties, resist dominant heterosexual scripts, and advocate for increased sexual minority rights. Sexual minority adolescents who “out” themselves through same-sex romantic relationships may thus find allies supportive of their identities and romantic relationships. Some evidence supports this possibility. Sexual minority adolescents who are “out” to heterosexual peers have larger peer networks compared to adolescents who do not disclose their sexual identity (Diamond & Lucas, 2004). Peer support for same-sex sexual identities and romantic relationships may also be increasing over time, so the friendship networks of contemporary same-sex youth may differ little from their heterosexual peers (Jones et al., 2022).

Gender may moderate associations between dating and number of friends for sexual minority adolescents, although the direction of these associations is not clear. Norms and stigma related to same-sex dating differ for boys versus girls. Boys experience particular stigma from other boys for same-sex attraction or insufficient displays of heterosexual interest (Bucchianeri et al., 2016; Pascoe, 2005). As a result, boys may experience more negative social consequences for same-sex dating than girls do. However, boys may also be more likely than girls to find allies in their female peers when they begin dating. Heterosexual girls play a visible role in allyship networks in high schools (Levesque, 2019), and their allyship focuses more often on sexual minority boys than girls. Cross-orientation friendships are more likely to be between heterosexual girls and sexual minority boys than any other constellation of gender and sexual orientation (Ueno, 2010). Cross-gender friendships with sexual minority boys may be desirable for heterosexual girls because girls do not need to adopt a sexual gatekeeper role in these relationships (Rose & Frieze, 1993; Ueno, 2010). Notably, there is a dearth of recent research on homophily and diversity in sexual minority youth’s friendships (Poteat et al., 2021).

## The rural school context

The present research focuses on a school-based sample of rural adolescents, a population of particular interest with respect to sexuality. School context may be particularly important for understanding sexual minority adolescents’ dating experiences. In-school dating relationships are especially visible to peers, which could mean that any social consequences of dating are heightened in school. Additionally, relationships with peers in school matter for sexual minority youth’s well-being (Poštuvan et al., 2019; Russell & Fish, 2016), although in one study number of friends specifically was not linked to mental health (la Roi et al., 2020).

Rural schools also have unique attributes that may matter for social experiences. Compared to urban or suburban contexts, schools in rural communities are likely to be smaller, less racially and ethnically diverse, and have students from lower socioeconomic backgrounds (Echazarra & Radinger, 2019). Additionally, youth in these communities are more likely to be exposed to less tolerant attitudes, increasing the likelihood of peer stigmatization of sexual minority youth and same-sex romantic relationships (Kosciw et al., 2015; Swank et al., 2012).

## The present research

We examine within-person associations between dating and number of friends for adolescents with same-sex and other-sex dating partners using longitudinal sociometric data from the PROSPER study of rural adolescents. Consistent with past research, we hypothesize that adolescents will experience more friendships, as measured by friendship nominations received, at times they have an other-sex dating partner, compared to times they are single. We hypothesize that dating will also be associated with changes in the number of friends for adolescents with same-sex partners, although the direction of this association is not clear. Because of the sexual double standard and gender homophily in friendship networks, we explore these associations separately for boys and girls, and for male friends versus female friends.

The inclusion of adolescents with same-sex relationships is an important contribution to this research. Understanding the links between dating and number of friends for sexual minority adolescents is important from a developmental perspective. Romantic relationships facilitate independence, sexual exploration and satisfaction, and affiliation (Connolly et al., 2023); for sexual minority adolescents, these relationships are also an important component of sexual identity development (Glover et al., 2009). Despite the developmental importance of romantic relationships, research on normative developmental processes lags behind research on problematic outcomes, such as dating violence and sexual risk for sexual minority youth (Mustanski, 2015). Researchers have called for work that examines sexual minority peer and romantic relationships from a normative framework, aiming to understand typical developmental trajectories and areas of resilience (Poteat et al., 2021). Addressing links between dating and number of friends will inform understanding of how achieving normative developmental milestones may alter the structure of peer relationships.

## METHOD

### Participants and procedure

Participants come from the PROSPER study, a longitudinal randomized trial of the PROSPER community-university partnership-based system for the delivery of evidence-based preventive interventions targeting substance use prevention in rural communities (Spoth et al., 2004, 2007). PROSPER is a community-university prevention partnership following two successive cohorts of students from 28 rural communities in Iowa (*n* = 14) and Pennsylvania (*n* = 14), with 1300 to 5200 enrolled public-school students per community. All study procedures were approved by the supervising institutions’ institutional review boards. Participation rates ranged from 86% to 90% across waves for all eligible students, with an average of 87.2% participation and about 11,000 students responding at each wave. Enrollment in the study was open at each wave, drawing the sample from the entire student body on each occasion (Osgood et al., 2013).

The PROSPER study followed students in one grade at each school longitudinally; at baseline, all students in the study were in 5th grade. Participants completed school-based assessments between the 5th and 12th grades. We used data from waves four through eight of the study, annual surveys corresponding to 8th to 12th grade. We focused on these assessment waves because participants reported the names of dating partners beginning in 8th grade (wave four). PROSPER utilized a sociometric data collection design where students were surveyed in their classrooms and asked to name up to seven same-grade friends and one romantic partner at school. To be eligible for the present analyses, which focus on within-person change, participants must have reported a dating partner in at least one wave and been single in at least one wave. Thus, we excluded approximately 9000 of the 14,000 study participants who provided data between waves four and eight because they did not report a matched romantic partner at any wave (although some reported a romantic partner in a different grade or school) and 74 because they did not report any friends at any wave of data collection. We also excluded approximately 2200 participants because they did not have variation in their relationship status—they completed only one wave of data collection or they were single or in a relationship at all waves of data collection. During data collection, coders identified unlikely people who were nominated as friends or romantic partners (e.g., celebrities, joke names) and removed these nominations from the dataset. In addition, coders reviewed survey responses for outliers during the data entry process. The final sample included 2826 adolescents and 9206 measurement occasions, with 2 to 5 measurement occasions per person.

Fifty-five percent of the participants were female. Regarding race and ethnicity, 87% of participants were White, 5% were Hispanic, 3% reported multiple races or a race not listed, 2% were Black, 1% were Asian, and less than 1% were Native American. Participants’ average age at W4 was 14.27 (SD = 0.39). The PROSPER study did not ask questions about sexual orientation or sexual identity during in-school data collection.

### Measures

Table 1 includes descriptive statistics pooled across waves and split by participant sex.

### Number of friends (friendship indegree)

At each wave of the study, participants responded to the question, “Who are your best and closest friends in your grade? Spell out the names the best you can.” Participants could list up to seven friends (two best friends and five additional close friends). Number of friends was measured by indegree (the number of classmates who named the participant as a friend). If someone the participant reported as a romantic partner reported the participant as a friend, this person was removed from the indegree count to ensure that any association between friendship and dating was not a methodological artifact of daters listing their partner as a friend. Of 162 cases where participants reported a same-sex romantic partner, in 35% of cases (*n* = 56), the person whom the participant nominated as a romantic partner nominated the participant as a friend. Of 4050 cases where participants reported an other-sex romantic partner, in 16% of cases (*n* = 635), the person whom the participant nominated as a romantic partner nominated the participant as a friend.

In inferential analyses, number of friends was standardized within the school and wave to better capture social standing relative to others in their class. We chose to standardize the number of friendships because differences in class sizes between schools and over time may influence the average number of received friendship nominations. By standardizing the measure, we reduce the likelihood that our outcome, number of friends, is confounded with the size of the pool of potential friends.

### Matched dating partners

Participants reported the name of their “current or most recent boyfriend or girlfriend, if [they] had any within the past year.” Matched dating partners were romantic partners who could be matched to another within-grade study participant. Participants reported their sex by responding to the question, “Are you…” with response options of “male” or “female.” Participants did not describe their partner’s sex, but because all partners were participants in the study the partners had reported their sex in their surveys. Based on the reported sex of the participant and the partner, we determined whether the romantic partner was a same-sex partner or other-sex partner. Of the 2826 participants (9206 person-waves) in the present research, 148 reported at least one same-sex dating partner (162 person-waves); 2728 reported at least one other-sex partner (4050 person-waves); and 50 reported both same-sex and other-sex partners. There were 4995 person-waves in which participants did not report a dating partner. We describe patterns of indegree by dating status in the Results section.

### Control variables

Grade level, race/ethnicity, grades in school, family structure, and free/reduced lunch (a proxy for socioeconomic status) were control variables. Each of these variables has been shown to be associated with number of friendships, sexual orientation, or both (Arias et al., 2018; Bos et al., 2008; Cheadle et al., 2015; Kreager et al., 2016). Linear and quadratic terms for *grade level* were used and coded such that 8th grade = 0. For *race/ethnicity*, two dichotomous variables indicated whether the participant reported Black or Hispanic identities. Because so few participants reported other races, we were unable to control for these identities in analyses. For *grades in school*, in each wave, participants reported their average grade in school, which was coded such that F = 0 and A = 4. For *family structure*, in each wave, students answered the question, “Who do you live with most of the year?” Students who responded that they lived with both parets, or a parent and a stepparent, were coded as living in a two-parent family (1). Students who responded that they live with only their mother, only their father, or in another type of household were coded as not living in a two-parent family (0). For *free/reduced lunch*, each wave, students answered the question, “What do you usually do for lunch on school days?” Students who responded that they receive free lunch from school or that they buy their lunch at school at a reduced price were coded as receiving free/reduced price lunch (1). Students who responded that they bring a lunch from home, go home for lunch, buy school lunch at full price, buy lunch outside of school, or do not eat lunch were coded as not receiving free/reduced price lunch (0).

### Analysis strategy

We examined within-person associations between matched romantic partnerships and a number of friends using three-level linear random effects models with time points (Level 1) nested within individuals (Level 2) nested within schools (Level 3). Time-varying effects included having a same-sex dating partner, having an other-sex dating partner, grade level, squared grade level, grades in school, whether the participant was in a two-parent household, and whether the participant received free/reduced price lunch. Person-level effects included participants’ sex, whether they were Black, and whether they were Hispanic. We also included person-level effects of having a same-sex dating and other-sex dating partner, which were operationalized as the proportion of waves included in the analyses in which the participant had a same-sex dating partner and the proportion of waves they had an other-sex dating partner. Finally, we included interactions of participant sex by within-person dating status. Separate models assessed the outcomes of standardized number of female friends and number of male friends. We present analyses conducted separately for boys and girls for ease of interpretation. In additional models not presented, we conducted the same analyses for all participants with gender as a predictor and participant sex × same-sex partner and participant sex × other-sex partner as interaction variables. In the results section, we note where significant interactions indicated different associations between dating and number of friends for boys versus girls. In all models, we utilized robust standard errors. The analyses only included waves when the participant had a matched partner and waves when the participant was single. Waves when the participant had a romantic partner not matched to another study participant were excluded from analyses. Analyses were conducted using the lmer package in R (Bates et al., 2015).

## RESULTS

### Descriptive statistics

Table 2 shows the average indegree by dating status for male and female adolescents. Separate rows are presented for participants who reported an other-sex partner at any point during the study and participants who reported a same-sex partner at any point.

### Multilevel models

**FIGURE 1:**
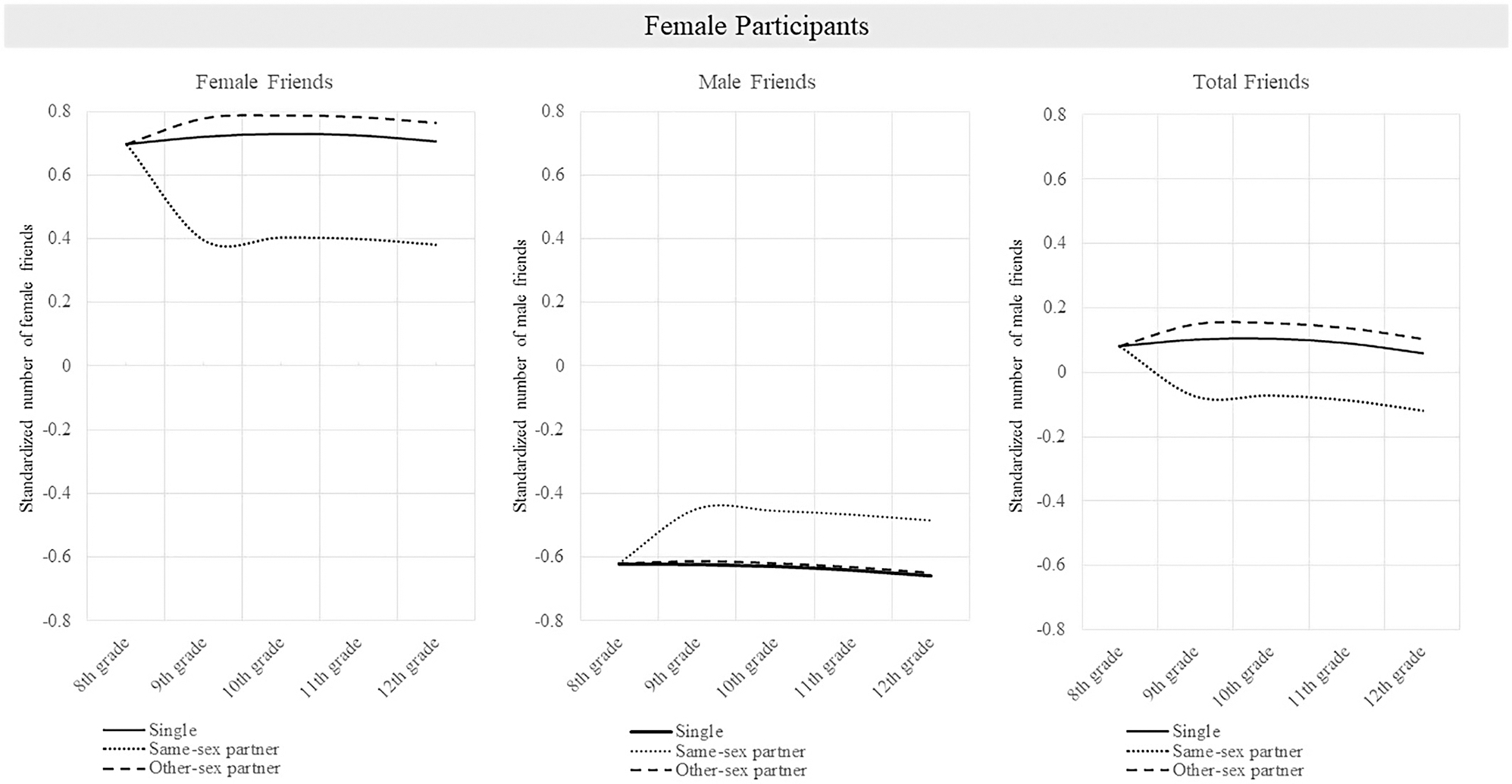
Predicted standardized friendship indegree by wave, and dating status for female participants. *Note*: This figure shows the predicted standardized friendship indegree for individuals who were single in 8th grade and either remained single, had a same-sex partner throughout high school, or had an other-sex partner throughout high school.

**FIGURE 2:**
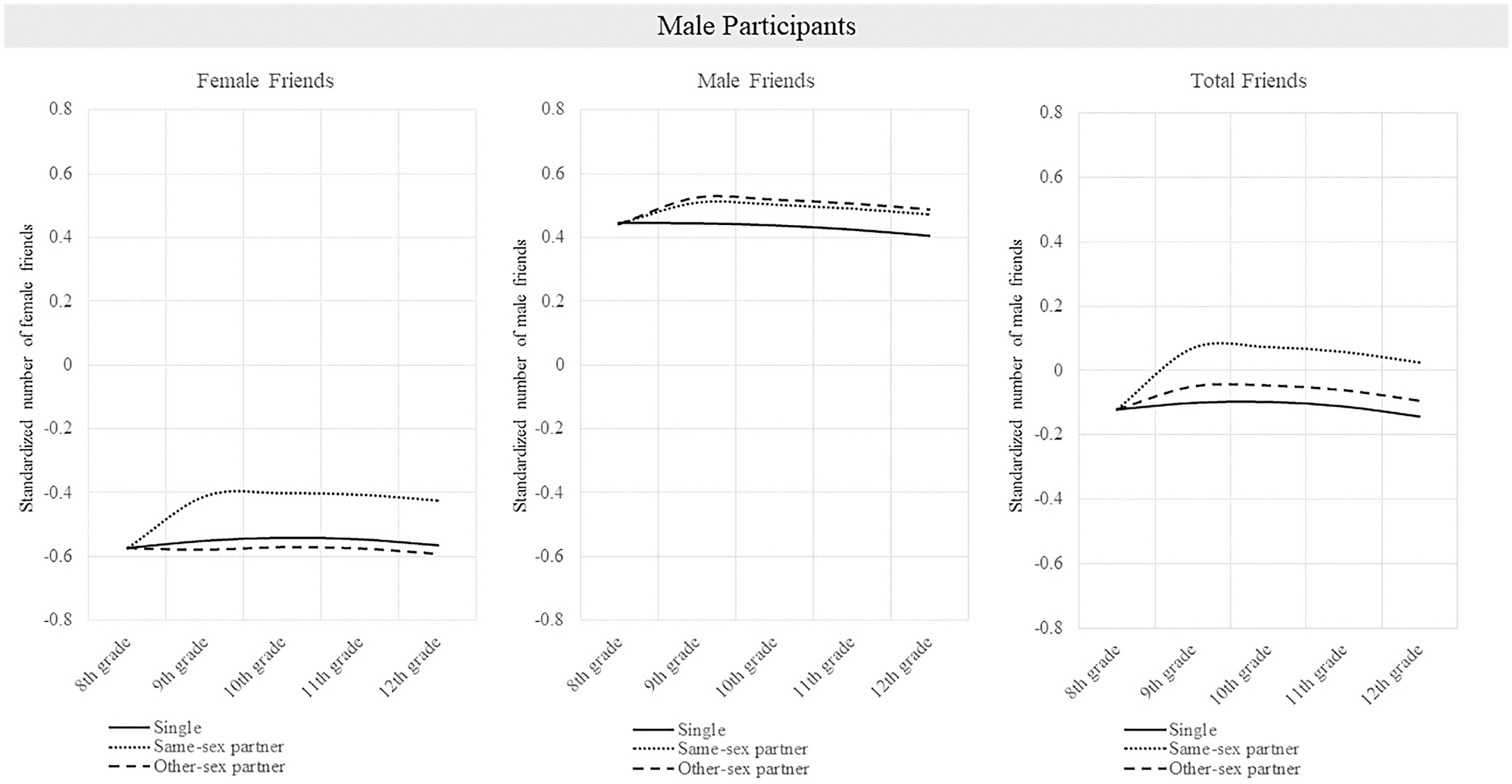
Predicted standardized friendship indegree by wave, and dating status for male participants. *Note*: This figure shows the predicted standardized friendship indegree for individuals who were single in 8th grade and either remained single, had a same-sex partner throughout high school, or had an other-sex partner throughout high school.

Results are displayed in Tables 3 (female participants) and 4 (male participants). Figures 1 and 2 display post-estimation predicted values. Regarding control variables, girls and boys reported fewer male and female friends when they received free or reduced price lunch, compared to when they did not (*p*’s range from <.001 to .016). Girls who lived in a two-parent household had more female friends (*p* = .004) and more total friends (*p* = .012), compared to girls with other family structures. Hispanic girls had fewer female friends (*p* = .050) and fewer total friends (*p* = .048), and Hispanic boys had fewer male friends (*p* < .001) and fewer total friends (*p* = .001), compared to students who were not Hispanic. Black boys had fewer male friends (*p* = .033) and fewer total friends (*p* = .014), compared to boys who were not Black.

### Same-sex dating

Girls had fewer female friends during waves they had a same-sex partner, compared to waves when they were single (*p* = .027). Because we used standardized values of indegree in analyses, all coefficients can be interpreted in terms of standard deviations. The coefficient of −0.26 for this effect means that girls’ number of friends was reduced by .26 SDs relative to others in their grade when they dated a same-sex classmate. On average, this corresponds to 0.62 fewer friends.

Girls had more male friends during waves they had a same-sex partner, compared to waves when they were single (*p* = .003, average of 0.11 more friends). Girls’ total number of friends did not differ significantly during waves they had a same-sex partner, compared to waves when they were single (*p* = .243). Boys had more female friends when they had a same-sex romantic partner, compared to when they were single (*p* = .022, average of 0.11 more friends). Boys’ number of male friends did not differ during waves they had a same-sex partner, compared to waves when they were single (*p* = .400). (Based on the gender × same-sex partner term in an equivalent model conducted with both male and female participants, the association between same-sex dating and number of female friends was significantly different for boys compared with girls.) Boys’ total number of friends did not differ significantly during waves they had a same-sex partner, compared to waves when they were single (*p* = .131).

One between-person effect of same-sex dating status was significant. Boys who spent more waves in a same-sex relationship had more total friends, compared to boys who spent more waves single (*p* = .050). Between-person effects of same-sex dating were not significant for boys’ number of female friends or male friends, however.

### Other-sex dating

Girls had more female friends during waves they had an other-sex partner, compared to waves when they were single (*p* = .013, average of 0.14 more friends). Girls’ number of male friends did not differ during waves they had an other-sex partner, compared to waves when they were single (*p* = .303). Girls had more total friends during waves they had an other-sex partner, compared to waves when they were single (*p* = .028, average of 0.13 more friends). Boys’ number of female friends did not differ when they had an other-sex partner, compared to when they were single (*p* = .054). Boys had more male friends when they had an other-sex partner, compared to waves when they were single (*p* = .002, average of 0.17 more friends). (Based on the gender × other-sex partner term in equivalent models conducted with both male and female participants, the associations of mixed-sex dating with number of female and male friends were significantly different for boys compared with girls.) Boys had more total friends during waves they had an other-sex partner, compared to waves when they were single (*p* = .026, average of 0.14 more friends).

One between-person effect of other-sex dating was significant for girls. Girls who spent more waves in an other-sex relationship had more total friends, compared to girls who spent more waves single (*p* = .043). Between-person effects of same-sex dating were not significant for girls’ number of female friends or male friends, however.

All between-person effects of other-sex dating were significant for boys. Boys who spent more waves in an other-sex relationship had more male, female, and total friends, compared to boys who spent more waves single (*p*’s range from .003 to <.001).

## DISCUSSION

Results suggest that dating has different associations with friendship network size for adolescents with same-sex partners versus other-sex partners. When adolescents had other-sex romantic relationships, they had more same-sex friends and more friends overall, suggesting that other-sex dating is socially rewarded by same-sex peers. Boys had more female friends when they were in a same-sex romantic relationship; in contrast, girls lost female friends and gained male friends when they were in a same-sex romantic relationship. Adolescents’ overall number of friends did not change when they had a same-sex relationship, compared to when they were single. These results have implications for understanding romantic development for sexual minority adolescents.

### Number of friends and heterosexual dating

Adolescents’ number of same-sex friends and overall friends increased when they had an other-sex partner, compared to times when they were single. This finding is consistent with past research, which has demonstrated associations between dating and being well-liked by peers (Furman et al., 2009; Furman & Rose, 2015; Miller et al., 2009; Ryjova et al., 2021; Savickaitė et al., 2019). Forming other-sex romantic relationships is a common, culturally accepted event that aligns with heteronormative cultural scripts about adolescent development and relationship formation, such that other-sex dating acts as a signal of social success and peer attractiveness.

Although we found that other-sex dating was linked to more same-sex friendships for boys and girls, it is interesting and unexpected that other-sex dating showed no associations with number of other-sex friendships. Prior research suggests that other-sex dating can be a mechanism for mixed-sex peer group formation common to adolescence (Dunphy, 1963) and create bridges for cross-sex peer influence (Kreager & Haynie, 2011; Wesche et al., 2019), yet we found little evidence that other-sex dating increases other-sex friendships. One possibility for this discrepancy is that, although dating adolescents are more popular with other-sex peers (Savickaitė et al., 2019), these other-sex peers may not become friends. Past research indicates that adolescent boys and girls have different friendship values; for example, girls place more emphasis on intimacy and boys place more emphasis on enjoyment (Rudolph & Dodson, 2022). These different values may hamper other-sex friendship formation.

### Same-sex dating is associated with more other-sex friends

Boys and girls had more other-sex friends when they had a same-sex partner, compared to when they were single. This finding is consistent with past research on young adults, which found that lesbian women and gay men were more likely to have an other-gender best friend than heterosexual participants were (Baiocco et al., 2014). These findings may reflect the gendered nature of friendship and allyship in adolescence.

First, a lack of potential sexual interest may open doors to friendship between heterosexual girls and sexual minority boys, and between heterosexual boys and sexual minority girls. In a heteronormative society, the expectation of sexual or romantic involvement can create unease in other-sex friendships (Linek, 2021). Without the potential for romantic conflict, forming other-sex friendships may be easier (Ueno, 2010).

Second, dating may lead to a restructuring of adolescents’ peer networks to meet developing friendship needs. Notably, boys and girls did not differ in their overall number of friends when they had a same-sex partner, compared to when they were single. Instead, our results suggest that their friendship networks became more composed of other-sex friends. In a study of Hispanic adolescents, Stanton-Salazar and Spina (2005) found that other-gender friends served a special purpose of being confidants, characterized by intimate counsel while being free of flirtation or sexual pursuit. For sexual minority adolescents in romantic relationships, other-sex friends may be especially important confidants. Societal homophobia could prevent same-sex heterosexual friends from providing social support for fear of being labeled as gay or lesbian themselves (Bortolin, 2010; Worthen, 2014). Other-sex friendships are not constrained by the same boundaries.

Third, having a romantic partner in their grade at school substantially increases sexual minority adolescents’ visibility as minorities, which opens them to experience the support of allies (Diamond & Lucas, 2004). Heterosexual girls are more likely than boys to belong to gay-straight alliance groups at their schools (Levesque, 2019), and their allyship in these spaces tends to focus more on sexual minority boys than sexual minority girls, sometimes described as adopting a “gay best (boy) friend” (Levesque, 2019). Our finding that boys have more female friends when they date a same-sex peer may reflect this phenomenon.

### Girls’ same-sex dating is associated with fewer female friends

Girls had fewer female friends when they had a same-sex partner, compared to when they were single. These findings may indicate that sexual minority girls face more negative social consequences of dating than boys do, given the gendered nature of allyship. In her ethnographic study of allyship in a high school gay-straight alliance, Levesque (2019) found that heterosexual girls distanced themselves from sexual minority girls in order to maintain their appearance of heterosexuality. The sexual minority girls in our sample may have experienced a similar phenomenon—as their outness increased due to forming in-school romantic relationships, it became riskier for heterosexual female peers to be friends with them.

Other possible explanations for this finding also exist. As girls form same-sex romantic relationships, their friendship needs may change. Research with primarily heterosexual samples indicates that in mid-to-late adolescence, girls may experience more social support and caregiving from their romantic relationships than boys do (Seiffge-Krenke, 2017). If this finding extends to sexual minority girls, they may need less support from other female friends when they are in romantic relationships, compared to times when they are single. As a result, they may restructure their friendships to be less focused on intimacy and more focused on enjoyment and recreation—values more often found in friendships with boys (Rudolph & Dodson, 2022).

### Boys’ same-sex dating is not associated with number of male friends

Interestingly, we did not find that adolescents lost male friends when they dated a same-sex partner. Therefore, our results do not support the idea that same-sex dating is stigmatized by adolescent boys. This finding is unexpected given the strong schemas that equate masculinity with heterosexuality (Pascoe, 2005; Phillips, 2005), which would lead heterosexual boys to reject sexual minority peers in order to maintain their appearance of masculinity. One possible explanation for this finding is that adolescents may not be “out” to everyone in their social circles, and male friends are not aware of boys’ same-sex relationships. Another explanation is that norms for masculinity are changing such that friendships between sexual minority and heterosexual boys are not as stigmatized as they were previously (Davis & Mehta, 2022; Magrath & McCormack, 2022). A third explanation is that sexual minority boys choose friends who are accepting of their sexual identities or share their identities. They may not lose friends when they date because their friends already know and accept their sexuality. Future research exploring friendship homophily in sexual orientation, as Poteat et al. (2021) call for, would help uncover whether this is the case.

Statistical power is a concern in our study, given the low numbers of participants who reported a same-sex dating relationship; therefore, we hesitate to make strong conclusions about links between same-sex dating and male friendships. More research is needed to understand whether the association we found amounts to adolescent boys’ indifference toward same-sex dating and if so, why this is the case.

### Limitations

The findings of the present research contain limitations, which can inform future studies. Although our use of within-person analyses rules out some confounding factors, we cannot make causal claims about the association between dating and number of friends. For example, it is possible that dating does not lead to more friends for heterosexual adolescents, but that these adolescents are more likely to seek out romantic partners at times when they are more well-liked and comfortable in their social networks. Additional research with more frequent measurement occasions could better address the temporal order of the association between dating and friendship network size.

Several measurement limitations may have influenced the validity of our results. First, this study focused only on romantic relationships among adolescents in the same grade and school. This strategy allowed us to examine romantic relationships that were especially visible to the peer network. However, out-of-grade and out-of-school romantic relationships could also affect adolescents’ social status, and we were unable to account for these in our analyses. Because age gaps are common in teen dating relationships (Koon-Magnin et al., 2010; Meier et al., 2016), additional research is needed to understand how out-of-grade dating may influence friendships for adolescents with same-sex and other-sex romantic partners.

Second, we have limited information about adolescents’ sexual identities and partnerships. Sexual orientation and identity were not measured in the study. Although sexual orientation and identity do not necessarily correspond to a romantic partner’s sex (Van Anders, 2015), knowing this information would help us understand the complexity of adolescents’ same-sex dating experiences. Knowing sexual orientation and identity could also help us understand whether adolescents were reporting their same-sex romantic partners authentically. Teenagers may falsely report a minoritized sexual identity as a joke (Robinson-Cimpian, 2014); similar “mischievous responding” may apply to reports of same-sex romantic relationships. The fact that we focused on same-sex partners who could be matched to another participant in the study lowers this possibility but does not eliminate it.

Third, we chose to measure status in peer groups via incoming friendship nominations. This strategy enabled us to understand how adolescents are perceived by their peers more objectively than self-report measures. However, being named as a friend is not the only component of peer relationships that indicates social integration, as Martin-Storey et al. (2015) discuss. In the future, researchers may consider how the quality of relationships and structure of friendship networks change as adolescents navigate their coming-out processes. For sexual minority adolescents in particular, social support and identity-related support matter for health (Doty et al., 2010; Watson et al., 2019). Being nominated as a friend indicates positive perceptions by others, but it could also represent a “gay best friend” phenomenon that tokenizes and isolates sexual minority youth from sources of social support (Levesque, 2019). In addition, it is unclear from these findings how same-sex and other-sex friendships matter for other aspects of well-being. For example, Zhang et al. (2015) found that same-sex friendships mattered more for boys’ loneliness than for girls’ loneliness. Additional research with other ways of operationalizing school experiences is needed to understand same-sex dating in adolescence.

The rural context of this study may have shaped our findings. Sexual minority adolescents in rural areas may be at increased risk of experiencing social isolation, compared to their urban peers (Kosciw et al., 2015; Swank et al., 2012). In urban environments, which tend to be more accepting of sexual minority youth, adolescents with same-sex dating partners may enjoy the same increase in number of friends that adolescents with other-sex partners experience. Additional research with representative samples is needed to clarify how location influences adolescents’ social experiences when dating.

## IMPLICATIONS AND CONCLUSION

Despite its limitations, this research offers important insights into the peer context of dating for sexual minority adolescents. As they age, sexual minority youth face the normative developmental milestone of forming romantic relationships, but with the twist of potentially making themselves vulnerable to social stigma due to their minoritized identity. Our findings suggest that adolescents with same-sex partners do not garner the same social benefits of dating that adolescents with other-sex partners may receive. However, sexual minority adolescents do not lose friends when they date a same-sex partner, which may represent resilience as they achieve important developmental milestones.

These findings have implications for understanding how sexual minority adolescents maintain interpersonal and community recognition as they navigate romantic development. In rural environments, such as the one in this study, sexual and gender minority youth report that visibility is key to well-being (Paceley et al., 2018). Because the composition of friendships is reorganized to focus more on other-sex friends, other-sex friends may be important allies for sexual minority adolescents who allow them to be “seen.” Social integration efforts may attend to building these other-sex friendships. In addition, as Levesque (2019) suggests, challenging schemas of masculinity and femininity may make it easier for sexual minority adolescents to be accepted by same-sex peers.

This research adds to a body of knowledge on how dating serves as a risk and/or protective factor for adolescents’ well-being. For adolescents in mixed-sex relationships, our findings add to existing research indicating that dating is associated with positive social outcomes (Furman et al., 2009; Furman & Rose, 2015; Miller et al., 2009; Ryjova et al., 2021; Savickaitė et al., 2019). For sexual minority adolescents, our findings expand on past research indicating that dating is protective against depression, alcohol use, and illicit drug use (Whitton et al., 2018a, 2018b). Our research suggests that dating may also lead to a reorganization of friendships with more emphasis on other-sex friends. Because of the protective effects of dating, relationship education programs are needed to help both heterosexual and sexual minority adolescents integrate dating into their lives in a healthy way. These programs must be sensitive to gender and sexual identity, given the distinct association between dating and number of friends for sexual minority boys versus girls.

## Figures and Tables

**TABLE 1:** Descriptive statistics by participant sex.

	Mean (SD) or proportion	
	Female participants	Male participants

Indegree (female friends)^a^	3.54 (2.40)	0.49 (0.95)
Indegree (male friends)^a^	0.42 (0.77)	2.90 (2.48)
Total indegree^a^	3.96 (2.61)	3.39 (2.70)
Same-sex dating partner^a^	0.02	0.02
Other-sex dating partner^a^	0.45	0.43
Black	0.02	0.03
Hispanic	0.06	0.05
Grades^a^	3.29 (0.79)	2.98 (0.92)
Two-parent family^a^	0.81	0.81
Free/reduced lunch^a^	0.17	0.17

*Note:* Raw indegree is presented here for ease of interpretation. Standardized values were used in multilevel models.

a Time-varying descriptive statistics are presented pooled across waves.

**TABLE 2:** Average indegree by dating status.

	Female participants		Male participants	
	Waves when single	Waves with a dating partner	Waves when single	Waves with a dating partner

Same-sex partner				
Female friends	4.02	2.33	0.26	0.61
Male friends	0.30	0.51	3.41	2.63
Total friends	4.33	2.83	3.67	3.27
Other-sex partner				
Female friends	4.09	3.67	0.44	0.51
Male friends	0.40	0.45	3.15	3.13
Total friends	4.49	4.11	3.59	3.64

*Note*: Raw indegree is presented here for ease of interpretation. Standardized values were used in multilevel models. Same-sex partner = adolescents who reported a same-sex partner at any wave during the study. Other-sex partner = adolescents who reported an other-sex partner at any wave during the study.

**TABLE 3:** Multilevel models of standardized friendship indegree, female participants (*N* = 4021 observations from 1271 participants).

	Female friends		Male friends		Total friends	
Predictors	Estimate	Std. error	Estimate	Std. error	Estimate	Std. error

(Intercept)	0.15	0.11	−0.71***	0.04	−0.41***	0.10
Same-sex partner	−0.26*	0.12	0.14**	0.05	−0.13	0.11
Other-sex partner	0.06*	0.03	0.01	0.01	0.05*	0.03
Grade level (8th grade = 0)	−4.63***	0.81	2.71***	0.33	−2.81***	0.78
Grade level squared	−2.08**	0.80	−0.90**	0.33	−2.29**	0.77
Grades in school	0.11***	0.02	0.03**	0.01	0.11***	0.02
Two-parent household	0.14**	0.05	0.01	0.02	0.11*	0.05
Free/reduced price lunch	−0.16***	0.05	−0.06**	0.02	−0.21***	0.04
Hispanic	−0.18*	0.09	−0.02	0.03	−0.17*	0.09
Black	0.06	0.16	0.02	0.05	0.05	0.15
Proportion of waves with same-sex partner	0.02	0.31	−0.01	0.11	0.07	0.29
Proportion of waves with other-sex partner	0.19	0.13	0.07	0.05	0.25*	0.13
Study condition (intervention = 1)	0.06	0.05	−0.06**	0.02	−0.10*	0.05
Random effects						
Participant	0.57		0.10		0.53	
School	0.37		0.03		0.33	
Residual	0.01		<0.01		0.01	
ICC: Participant	0.39		0.24		0.38	
ICC: School	0.01		0.02		0.01	

*Note*:

* *p* < .05.

** *p* < .01.

*** *p* < .001.

**TABLE 4:** Multilevel models of standardized friendship indegree, male participants (*N* = 5185 observations from 1555 participants).

Predictors	Female friends		Male friends		Total friends	
	Estimate	Std. error	Estimate	Std. error	Estimate	Std. error

(Intercept)	−0.75***	0.04	0.06	0.09	−0.68***	0.08
Same-sex partner	0.10*	0.05	0.08	0.09	0.14	0.09
Other-sex partner	−0.02	0.01	0.07**	0.02	0.05*	0.02
Grade level (8th grade = 0)	4.59***	0.37	−3.94***	0.77	1.63*	0.76
Grade level squared	0.14	0.36	0.33	0.75	0.04	0.74
Grades in school	0.02*	0.01	0.08***	0.02	0.09***	0.02
Two-parent household	0.02	0.02	0.02	0.04	0.03	0.04
Free/reduced price lunch	0.03	0.02	−0.06	0.04	−0.03	0.04
Hispanic	<0.01	0.04	−0.37***	0.10	−0.31**	0.10
Black	−0.06	0.06	−0.30*	0.14	−0.33*	0.13
Proportion of waves with same-sex partner	0.03	0.13	0.56	0.31	0.57*	0.29
Proportion of waves with other-sex partner	0.19***	0.05	0.38**	0.13	0.50***	0.12
Study condition (intervention = 1)	0.04*	0.02	−0.04	0.05	0.08	0.05
Random effects						
Participant	0.12		0.50		0.50	
School	0.07		0.56		0.46	
Residual	<0.01		0.01		0.01	
ICC: Participant	0.38		0.52		0.48	
ICC: School	0.01		0.01		0.01	

*Note*:

* *p* < .05.

** *p* < .01.

*** *p* < .001.
